# The Molecular Mechanisms of Plant-Derived Compounds Targeting Brain Cancer

**DOI:** 10.3390/ijms19020395

**Published:** 2018-01-30

**Authors:** Hueng-Chuen Fan, Ching-Shiang Chi, Yu-Kang Chang, Min-Che Tung, Shinn-Zong Lin, Horng-Jyh Harn

**Affiliations:** 1 Department of Pediatrics, Tung’s Taichung Metroharbor Hospital, Wuchi, Taichung 435, Taiwan; fanhuengchuen@yahoo.com.tw (H.-C.F.); chi-cs@hotmail.com (C.-S.C.); 2 Department of Rehabilitation, Jen-Teh Junior College of Medicine, Nursing and Management, Miaoli 356, Taiwan; yogurt8306@gmail.com; 3 Department of Medical Research, Tung’s Taichung Metroharbor Hospital, Wuchi, Taichung 435, Taiwan; 4 Department of Surgery, Tung’s Taichung Metroharbor Hospital, Wuchi, Taichung 435, Taiwan; tungminche@yahoo.com.tw; 5 Buddhist Tzu Chi Bioinnovation Center, Tzu Chi Foundation, Hualien 970, Taiwan; shinnzong@yahoo.com.tw; 6 Department of Neurosurgery, Buddhist Tzu Chi General Hospital, Hualien 970, Taiwan; 7 Department of Pathology, Buddhist Tzu Chi General Hospital and Tzu Chi University, Hualien 970, Taiwan

**Keywords:** blood-brain barrier (BBB), *n*-butylidenephthalide (BP), glioblastoma multiforme (GBM), isochaihulactone (ICL), *O*-6-methylguanine-DNA methyltransferase (MGMT), protein kinase C (PKC), retinoblastoma protein (Rb), temozolomide (TMZ)

## Abstract

Glioblastoma multiforme (GBM) is one of the most aggressive and malignant forms of brain tumors. Despite recent advances in operative and postoperative treatments, it is almost impossible to perform complete resection of these tumors owing to their invasive and diffuse nature. Several natural plant-derived products, however, have been demonstrated to have promising therapeutic effects, such that they may serve as resources for anticancer drug discovery. The therapeutic effects of one such plant product, *n*-butylidenephthalide (BP), are wide-ranging in nature, including impacts on cancer cell apoptosis, cell cycle arrest, and cancer cell senescence. The compound also exhibits a relatively high level of penetration through the blood-brain barrier (BBB). Taken together, its actions have been shown to have anti-proliferative, anti-chemoresistance, anti-invasion, anti-migration, and anti-dissemination effects against GBM. In addition, a local drug delivery system for the subcutaneous and intracranial implantation of BP wafers that significantly reduce tumor size in xenograft models, as well as orthotopic and spontaneous brain tumors in animal models, has been developed. Isochaihulactone (ICL), another kind of plant product, possesses a broad spectrum of pharmacological activities, including impacts on cancer cell apoptosis and cell cycle arrest, as well as anti-proliferative and anti-chemoresistance effects. Furthermore, these actions have been specifically shown to have cancer-fighting effects on GBM. In short, the results of various studies reviewed herein have provided substantial evidence indicating that BP and ICH are promising novel anticancer compounds with good potential for clinical applications.

## 1. Introduction

Brain tumors, which are a main cause of morbidity and mortality in human being, often cause severe disabilities and heavy burden in families and health care systems. The incidence estimates 25.48 per 100,000 [1]. Among them, glioblastoma multiforme (GBM) is one of the most aggressive and malignant forms of brain tumors. Moreover, GBM accounts for approximately 20% of all brain tumors [2]. In the US, the incidence of GBM is 3.19 per 100,000 people per year [3], while in Taiwan, the incidence is 0.85 per 100,000 people per year [4].

Even with the application of maximal surgical excision plus radiotherapy along with concomitant and/or subsequent chemotherapy, the relative survival estimates for GBM are extremely low. Specifically, the mean survival time for patients newly diagnosed with GBM is only 12 to 15 months, with a 2-year survival rate of only 13–26% and a 5-year survival rate of less than 5% [5]. In short, the prognosis for patients with this highly malignant form of tumor remains dismal, with the invasive and diffuse nature of brain tumors being one of the key factors accounting for the high rate of deaths from these cancers relative to their incidence [6].

A variety of chemotherapy regimens may be worthy of a clinical trial, with the majority of these regimens consisting of high doses of a single drug or a combination of multiple drugs. As with surgery and radiotherapy, however, the efficacy of chemotherapy is usually limited for various reasons. Firstly, the penetration of chemotherapeutic drugs into the brain is typically incomplete because most drugs cannot easily cross the blood–brain barrier. Secondly, the cells in brain tumors are generally highly heterogeneous, such that if a given drug destroys some of the targeted cells, it may fail to extirpate others [7]. Thirdly, further issues limiting the efficacy of chemotherapy include toxicity and the fact that some of the initially responsive cancer cells may relapse and develop resistance to multiple anticancer agents with different structures and mechanisms of action, leading to dissemination and eventual death from the disease. Taken together, these reasons are largely responsible for the frequent limitations of and transitory benefits resulting from chemotherapy regimens [8,9,10,11,12]. Overall, the diffuse, invasive nature and locations of brain tumors typically mean that none of the standard treatments, including surgery, radiotherapy, or chemotherapy, are completely successful in destroying all the tumor cells. These limitations underscore the need for continuing investigations of novel and alternative therapeutic options, including clinical trials of any agents showing therapeutic potential.

Natural products, including some from plants used in traditional Chinese medicine and other forms of traditional medicine, such as *Angelica sinensis*, *Annona glabra*, *Bupleurum scorzonerifolium*, and *Bursera microphylla*, have shown promising therapeutic effects on brain cancers and other forms of cancer [13,14,15,16], as well as anti-degenerative effects on Alzeheimer’s disease, spinocerebellar ataxia, and amyotrophic lateral sclerosis [17,18,19]. The present review provides an overview of the research from 2000 to 2017 by the use of PubMed (https://www.ncbi.nlm.nih.gov/pubmed/) retrieving regarding the use of such extracts in targeting brain cancer, including the role of genetic and epigenetic factors in the benefits they provide.

## 2. The Past and Present Use of Natural Products as Cancer Therapies

It is important to note that natural (as opposed to synthetic) products, including plant extracts, have been used in cancer treatment for a long time, with folk remedies leading to the discovery of some of the most effective cancer treatments available in the past few decades. For example, a podophyllin-containing material, *Podophyllum peltatum* root extract, was traditionally utilized by American Indians to treat skin cancers and other conditions, a history which helped to inspire the search for more effective and less toxic podophyllotoxin derivatives. That search led in turn to the development of etoposide, which is currently used to treat GBM, as well as various other cancers [20,21].

Paclitaxel (Taxol) was firstly derived from the bark of the *Taxus brevifolia* tree as part of a large-scale screening program conducted by the National Cancer Institute. Following a long journey from its discovery in 1967 to its initial clinical usage, paclitaxel eventually went on to become the best-selling anticancer drug in the early 2000s thanks to its efficacy against breast, ovarian, and non-small cell lung cancers [22]. In a similar chronicle of drug development, the *Camptotheca acuminata* tree served as the source for camptothecin, derivatives of which (namely, irinotecan and tapotecan) have become mainstream treatments of colon cancer, small cell lung cancer, ovarian cancer, and cervical cancer [23].

In short, the use of natural compounds and their derivatives in cancer treatment has a long history and extensive present, with some of the most popular and effective cancer drugs available today having been derived from natural sources, including plants. As such, while using complementary and alternative medicine treatments, many of which are plant-based, is understandably viewed with some caution, or even skepticism, especially with respect to the treatment of cancer [24,25], clinicians should bear in mind that a number of chemotherapies derived from plants are entirely mainstream and supported by extensive clinical research. Relatedly, one aim of this review is to identify those plants and plant extracts which show genuine promise for the treatment of brain cancer, with our hope being that readers can better distinguish these more promising agents from other natural products that may lack much, if any, scientific evidence supporting their use.

## 3. Underlying Molecular Mechanisms for Natural Products Triggering the Death of Cancer Cells

It was mysterious that how a cell controls its life or death, but now it is gradually clear that there are several molecules and signaling pathways tightly controlling the life or death of a cell. Genes that are altered in neoplasia affect three major biologic pathways that normally regulate cell grow. There are a variety of molecular mechanisms of natural products that can trigger the death of cancer cells. In this review, however, we focus exclusively on those mechanisms relating to cell cycle machinery, apoptosis, and telomerase, as these are the most common means of inducing cancer cell death.

### 3.1. Cell Cycle Machinery

The cell cycle, one of the most dynamic processes in biology, drives the proliferation of a cell. A highly regulated process consisting primarily of the S phase (DNA synthesis) and M phase (mitosis), which are separated by two gap phases (G1 and G2), the cell cycle is tightly controlled by numerous regulatory proteins, including the cyclins, cyclin-dependent kinases (CDKs), their substrate proteins, the CDK inhibitors (CKIs), and the tumor-suppressor gene products p53 and pRb. The G1 to S transition is controlled by the p16-Rb pathway. In this pathway, P16 binds to CDK4/6, inhibiting its kinase activity and thereby preventing retinoblastoma protein (Rb) phosphorylation.

Rb is a crucial regulator of cellular senescence. Rb may bind to the transcription factor E2F1 to prevent from transcription of the E2F1 target genes that encode signals involved in DNA replication. Rb-p16-19Arf proteins and activated E2F can initiate premature cell senescence in a mouse pituitary tumor model [26]. The execution of cell cycle arrests by the formation of repressor complexes between the Rb family (pRb, p107 and p130) and E2F in the nucleus [26,27]. *Rb*, a tumor suppressor gene, suppresses human tumors by inhibiting p16 [28]. The phosphorylated Rb increases the expression of P16 to inhibit CDK4/6, leading to increased levels of hypophosphorylated Rb, which in turn leads to decreased p16 expression [29]. However, the levels of p16 expression do not correlate with the levels of phosphorylated Rb during the cell cycle [30]. In the process of aging, the increased expression of p16 is irrelevant to telomere shortening, but is associated with the exposure of radiation or DNA damaging agents [31,32]. The p16/Rb pathway in combination with the mitogenic molecules trigger the formation of reactive oxygen species (ROS), which consequently initiates the protein kinase C delta (PKC δ) in establishing stable G1 cell-cycle arrest, leading to cellular senescence [33]. p16 may have dual functions in the maintenance and initiation cellular senescence. Accordingly, control of p16 expression is an important mechanism for the suppression of senescence.

Those factors accelerate and brake the execution of the cell cycle to prevent the occurrence of any errors in the stages of cellular proliferation, replication, mitosis, etc. Briefly, it is also worth noting that in resting cells, the activities of CDKs are either low or completely halted. During such periods, the non-phosphorylated Rb binds and inhibits the E2F transcription factor. Cell-cycle entry can then be activated when mitogenic signals are transmitted to resting cells, which are activated through the Erk-dependent activation of transcription factors, such as the c-Myc and V-ets erythroblastosis virus E26 oncogene homolog 1 (ETS-1), which in turn up-regulate cyclin D. Cyclin D then binds its cognate CDKs, CDK4 and CDK6, to initiate the phosphorylation of Rb, which in turn liberates the E2F transcription factor and allows the cell to enter the S phase. CDK4 and CDK6 are inhibited by p16 and p27. The *p53* gene product is an important cell cycle check-point regulator at both the G1/S and G2/M check points. Acting as a DNA-binding transcription factor, p53 regulates specific target genes to arrest the cell cycle and initiate apoptosis. Following DNA damage, the CDKI p21 is expressed in a p53-dependent or p53-independent manner [34]. p21 inactivates the cyclin B1/cell division cycle 2 (CDC2) complex and disrupts the interaction between proliferating cell nuclear antigen and the cell division cycle 25C (CDC25C) to arrest cell cycle arrest at G2 phase [35]. P53 activates the transcription of several genes to control the cell cycle, including p16, p21, p27, GADD45, and MDM2. The accumulation of pRb induces an arrest of proliferation, which is overcome by the co-expression of cyclin A or E. It is well known that the transition from the G2 phase to mitosis is triggered by the cdc25-mediated activation of the cyclin B1/cdc2 complex and that cyclin B1/cdc2 activation is triggered by cdc25, a phosphatase that dephosphorylates Thr15 [36,37].

### 3.2. Apoptosis

In addition to inducing cell-cycle arrest, if cells are in a state of severe damage, such that they are beyond being repaired, they may induce their own death. Apoptosis, or the process of programmed cell death, is an important homeostatic mechanism that is characterized by unique morphological and biochemical features and is used maintain the appropriate numbers of cells in the body. There are several crucial cellular and molecular biological features involved in apoptosis, including cell shrinkage, the disorganization of chromatin, the externalization of phosphatidylserine, and the activation of a group of cysteine proteases named “caspases” that play a vital role in the initiation of the death signals leading to apoptosis [38]. Caspases can be divided into initiators, including caspase-1, -2, -8, -9 and -10, which are involved in early stages of the proteolytic cascade, and effector caspases, including caspase-3, -6 and -7,which are involved in the cleavage of specific intracellular substrates (e.g., poly-ADP-ribose polymerase, focal adhesion kinase). Consequently, complex cascades link the initial stimuli to the final demise of the cell [39]. A variety of physiological and pathological stimuli and conditions can trigger apoptosis, but not all cells are affected by the same stimuli [40]. In general, apoptosis keeps a balance between cell division and cell death, maintaining the appropriate numbers of cells in the body. Aberrant apoptosis, however, is observed in several types of cancer. As such, the manipulation of apoptosis in cancer cells, including via manipulations of p53, can serve as a potential strategy for anticancer drug development.

While an estimated 5–10% percent of cancer cases result from genetic defects inherited directly from parents of individuals, more typically, a number of genetic changes (such as mutations, chromosomal rearrangements, mitotic recombinations, etc.) must occur before a cancer begins to grow in a person [1]. A range of behavioral and environmental factors can generate these changes, but extensive epidemiological studies have implicated smoking, alcohol use, obesity, and low fruit and vegetable intake as the leading causes of cancer around the world [27].

Ongoing advances in the field of genetics in recent decades have even allowed researchers to identify the specific genes that lead to various cancers, as well as, in some cases, the precise mechanisms that change their genetic effects [41]. The *p53* tumor suppressor gene constitutes a particularly conspicuous example, as it is mutated in roughly half of all types of cancers originating in a wide range of bodily tissues, although hundreds of other cancer-related genes have also been identified [42,43]. In short, genes play a significant role in all cancers in one way or another, although it is possible to further distinguish between genetic abnormalities and functional genomic modifications that do not alter the nucleotide sequence itself. The latter type of alterations is referred to as epigenetic alterations.

### 3.3. Telomerase

While in cell division, the eukaryotic DNA polymerase protein complex cannot completely replicate the sequences at telomeres, the terminals of the chromosomes. Telomere shortening blocks cell division that cannot be repaired in normal somatic cells, causing damaged cell going into apoptosis. Therefore, telomere shortening may act as a genetic time bomb and mitotic clock, involving in chromosomal abnormalities in senescence, transformation, and immortalization [44].

Human telomerase, a ribonucleoprotein enzyme that composed telomerase RNA, a constitutively expressed template-containing RNA, is a crucial enzyme in replenishing the telomere length by capping hexameric (TTAGGG) repeats in the process of DNA replication. Interestingly, an extraordinarily high level telomerase activity (TA) is detected in most of human cancer specimens, whereas most somatic cells do not display or only at very low levels of TA. As the telomere length in nearly all tumor cells is stably maintained, telomere dysfunction and telomerase activation may possibly ignite the fuse of a growing cancer [45].

The telomerase reverse transcriptase (hTERT), a catalytic subunit bearing the enzymatic activity of telomerase, is a rate-limiting determinant of the enzymatic activity of human TA [46]. By using TRAP assay, we found TA was correlated expression levels of the human telomerase reverse-transcriptase (hTERT) mRNA isoforms in the hepatic cell carcinoma cells [47,48,49,50], suggesting that hTERT expression may be tightly associated with cancer-related telomerase activation, cellular senescence, immortalization, and carcinogenesis in humans. Importantly, the hTERT may be a sensitive indicator of telomerase function and activity for accessing the disease status and effects of therapeutic interventions.

In short, by screening patients for specific genetic and epigenetic abnormalities and then treating them with therapies specifically tailored to those abnormalities, researchers and clinicians are achieving more and more success against various types of cancer [51]. Note that among the many specific examples of such success, one with particular relevance to this review is the finding that GBM patients with a methylated *O*-6-methylguanine-DNA methyltransferase (MGMT) promoter gene are known to exhibit better response rates and prognosis when treated with temozolomide than patients without that specific abnormality [52].

## 4. The Use of Plant-Derived Compounds to Target Cancer via Genetic and Epigenetic Alterations

As noted above, plant extracts and their derivatives have long been used in cancer treatments, and many of those compounds achieve their cancer-fighting effects via genetic and epigenetic alterations. Etoposide, one the drugs ultimately derived from *Podophyllum peltatum* (commonly known as the wild mandrake), provides a prominent example. A type of topoisomerase inhibitor, etoposide targets human type IIA topoisomerases (Top2α and Top2β) in order to cause errors in DNA synthesis leading to the destruction of cancer cells [53]. More specifically, DNA topoisomerase enzymes are responsible for a number of key actions underlying the replication of both normal and cancer cells, including the separation of DNA strands for transcription and replication, the compacting of the genome within a cell, and the segregation within two daughter cells of identical copies of a replicated cell’s entire genome [54]. As such, by trapping the cleavage complexes of the type IIA topoisomerases, etoposide effectively blocks DNA re-ligation, thereby causing DNA strand breaks that, in turn, disrupt the entire process of cell replication and preferentially promote the apoptotic destruction of cancer cells [53,55].

Such direct targeting of cancer cell DNA constitutes one relatively common mechanism of action for chemotherapy, and in fact, several other plant-derived anticancer agents are also members of the topoisomerase inhibitor family of medications, effectively targeting cancer cells in manners quite similar to that utilized by etoposide. For example, irinotecan and toptecan, which are analogues of camptothecin, are topoisomerase I inhibitors that initiate the destruction of cancer cell DNA via mechanisms quite similar to that induced by etoposide [56]. In contrast, while also disrupting cancer cell replication in a manner ultimately leading to apoptosis, vinblastine and vincristine, which are derivatives of the *Madagascar Periwinkle* (*Catharanthus roseus*), accomplish their effects by binding to the tubulin protein. This capture of the tubulin within a given cell prevents the cell from undergoing the metaphase of mitosis in a proper manner, which in turn causes it to undergo apoptosis [57].

Unfortunately, while the aforementioned drugs have proven at least somewhat effective in fighting various cancers, causing their widespread usage, they are typically far from being curative. For example, one randomized phase III trial comparing etoposide combined with cisplatin to paclitaxel combined with carboplatin in the treatment of advanced or metastatic non-small cell lung cancer reported 1-year survival rates of only 37% and 32%, respectively, for the two treatment regimens [58]. Meanwhile, the prognoses for various forms of brain cancer remain dismal, the modest efficacy of some existing treatments notwithstanding. As such, the need for continued investigations of novel and alternative therapeutic options likewise remains.

## 5. Plant-Derived Compounds That Have Recently Exhibited Promise in Treating Brain Cancer

In recent years, various studies of plant extracts and derivatives, such as phyto-derivative BRM270 [59], genistein [60], biochanin A [60], resveratrol (trans-3,4′,5-trihydroxystilbene) [61], epigallocatechin gallate [62], retinoids [63], and cannabis and cannabinoids [64,65] have been conducted and shown potential effects against GBM. The use of δ 9-tetrahydrocannabinol (THC) in combination with Temozolomide (TMZ) has shown an effective anticancer activity through the activation of CB1 and CB2 to impair the proliferation of GBM [64] and a phase I clinical trial with a direct intratumoral injection of THC in recurring GBM has been safely conducted [66]. However, due to the page limits, we focus exclusively on two promising plant-derived compounds, the *n*-butylidenephthalide (BP) and isochaihulactone (ICL) and provide an overview of the evidence supporting their use and their apparent mechanisms against GBM.

### 5.1. Effects of Angelica Sinensis (AS) in Brain Cancer Therapy

#### 5.1.1. Penetration through the Blood-Brain Barrier (BBB)

*Angelica sinensis* (Oliv.) Diels (AS), commonly known as dong quai, is used in traditional Chinese medicine for a wide variety of conditions, including symptoms of menopause, gastric mucosal damage, impaired myocardial blood blow, hepatic injury, and chronic glomerulonephritis, suggesting that AS may possess a broad spectrum of pharmacological activities [67,68,69,70,71]. Our previous study provided one of the first investigations of its potential anti-tumor effects. Specifically, the chemotherapeutic effects of a chloroform extract of *A. sinensis* (AS-C) displayed impressive effects, suppressing the proliferation of glioblastoma multiform (GBM) cells both in vitro and in vivo through both p53-dependent and p53-independent pathways to induce apoptosis without cytotoxic effects on normal fibroblast cells. AS-C induced significant levels of p21 and p16, as well as decreased phosphorylation of Rb, causing arrest of the cell cycle. Additionally, Fas, a death receptor triggering the activation of procaspase-8 and procaspase-3 to initiate apoptosis, was found to exhibit increased expression after AS-C treatment. Notably, these results suggested that the AS-C exhibited relatively good ability to penetrate the BBB, a capacity which likely contributed substantially to its efficacy against GBM cells. In contrast, many compounds exhibit no or limited ability to penetrate the BBB, which is one of the main reasons that only a limited number of drugs are currently being developed to treat malignant brain tumors. As such, our data support the conclusion that AS-C could potentially serve as a source of potent compounds against GBM [13].

#### 5.1.2. Growth Arrest, Anti-Proliferation, and Apoptosis

In a subsequent study [72], we demonstrated the anti-GBM effects both in vitro and in vivo of one such compound, namely, *N*-butylidenephthalide (BP), which is itself isolated from AS-C. After treatment with BP, human GBM brain tumor cells were then analyzed by microarray screening. Among various BP-induced genes, we identified the Nuclear receptor subfamily 4 group A member 1 (NR4A1; Nurr77), which is known to be involved in the processes of growth arrest, anti-proliferation, and apoptosis [73]. We found that BP increased the mRNA and protein levels of nuclear receptor Nurr77 in a time-dependent and Activator protein 1 (AP1)-related manner. More specifically, BP initiated the translocation of Nurr77 from the nucleus to the cytoplasm, leading to the release of the cytochrome c from the mitochondria and the activation of caspase-3, resulting in apoptosis. Additionally, short interfering RNA (siRNA) Nurr77 blocked BP-induced apoptosis, supporting the conclusion that the anti-tumor effect of BP is mainly due to its targeting of Nurr77.

#### 5.1.3. Apoptosis and Senescence

To investigate the anti-GBM effect of BP in vivo, we incorporated the chemical into a biodegradable polyanhydride wafer, which is an intracranial drug delivery system used to eliminate a residual tumor. In that study, the wafers were surgically implanted into xenograft animal models, including F344 rats injected with rat GBM cells and nude mice injected with human GBM cells. Our results showed that the wafers containing BP delivered a sufficient concentration of BP to the tumor site without significant toxicity in the surrounding normal brain tissues. The BP-wafers exhibited a significant inhibitory effect on tumor growth without causing any significant adverse effects on the animals in a dose-dependent manner through three main mechanisms. First, BP decreased the expression of hTERT mRNA to cause tumor senescence. Secondly, BP activated the PKC pathway to increase Nurr77 transcription via an AP-1 motif, resulting in apoptosis. Third, BP induced the translocation of Nurr77 from the nucleus to the cytoplasm, leading to cytochrome c release and caspase-3-dependent apoptosis [74]. All of these findings support the substantial effect of BP against GBM.

#### 5.1.4. Anti-Chemoresistance

TMZ, a chemotherapeutic regime for GBM, is an alkylating agent which breaks the DNA double-strand, thus causing cell cycle arrest and ultimately cell death [75].Patients with GBM whose tumors have a methylated promoter for the gene encoding MGMT have previously been found to be more likely to benefit from the addition of TMZ [5]. However, high expression of MGMT in cancer cells may lead to blunting of the therapeutic effect of TMZ. Our data showed that BP possessed a combination effect with TMZ against GBM, reducing MGMT expression in order to overcome chemoresistance to TMZ in GBM cells [76].

#### 5.1.5. Anti-Invasion, Anti-Migration and Anti-Dissemination

**Figure 1:**
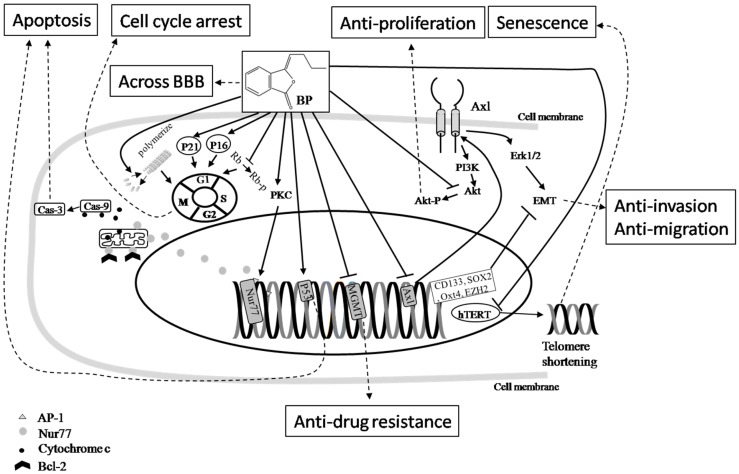
The cancer-fighting mechanisms of BP. The effects of BP in brain cancer therapy include impacts on cancer cell apoptosis, cell cycle arrest, senescence, and telomerase inactivation. The compound also exhibits a relatively high level of penetration through the blood-brain barrier (BBB). Taken together, these impacts and properties have been shown to be anti-proliferative, anti-drug resistance, anti-invasion, anti-migration, and anti-metastatic. Symbol key: 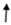 Pathway; 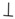 Inhibition; 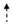 Final effect. Please see the content in Section 5.1.

As dissemination is the most important cause of morbidity and mortality in GBM at advanced stages [77], recent advances in cell biology have shown that the epithelial-to-mesenchymal transition (EMT), a reversible and dynamic process by which epithelial cells are converted into mesenchymal cells, triggers alterations in cell-cell adhesion and cell matrix degradation, as well as infrastructure changes to the cell membrane, through various complex signaling pathways, including integrin, tyrosine kinase, Wnt signaling, transforming growth factor-β receptor (TGF-βR), and Notch signaling pathways, to disperse cells in embryos, form mesenchymal cells in injured tissues, regulate embryonic stem cell differentiation, and initiate the invasive and metastatic behavior of cancers [78]. In one of our previous studies, following treatment with BP, human GBM brain tumor cells were analyzed by microarray screening. Among various BP-induced genes, we identified the *Axl* gene, which is a receptor tyrosine kinase linked to a variety of high-grade cancers and their poor prognoses [79]. Our data showed that the treatment of BP substantially inhibited the expression of Axl in a dose- and time-dependent manner through the extracellular signal regulated kinases pathway. Our data confirmed that Axl was a crucial target in the inhibition of GBM EMT-related genes, including *N*-cadherin, Twist, Snail, and Slug expression; the controlled-release BP wafer not only delivered a significant drug concentration but also extended the drug diffusion distance within the tumor cells, which in turn significantly increased the survival rate of the treated animals and reduced tumor invasion and migration through a decrease of Axl expression in the in vitro and in vivo xenograft animal models (Figure 1) [80].

#### 5.1.6. *N*-Butylidenephthalide (BP), a Promising New Anticancer Chemical

We advanced this work still further, providing greater details regarding the anti-GBM effects of BP and the means by which they are accomplished. For example, Enhancers of Zeste 2 (EZH2) and Axl, which are highly expressed in GBM [81,82], are involved in the immortalization, unlimited proliferation, blocking of apoptosis, development of chemoresistance, invasion, migration, and dissemination of GBM. One, some, or all of those malignant properties of GBM may contribute to its high rate of recurrence rate and poor prognosis. Our results demonstrated that overexpressed CD133/Axl/EZH2/TGF-β1 were significantly associated with tumor invasion, migration, and EMT. Those malignant features could be attenuated by treatment with BP resulting in a decrease of the expression of Axl and EMT-related genes in a dose-dependent manner [83]. These results indicated that BP is a promising new anticancer compound with excellent potential for clinical application.

### 5.2. Effects of Bupleurum scorzonerifolium in Brain Cancer Therapy

#### 5.2.1. Telomerase Inhibition and Apoptosis in Lung Cancer

The root of *Bupleurum scorzonerifolium*, known as Nan-Chai-Hu in Chinese, has traditionally been used in China, Japan, and other parts of Asia to treat hepatitis, cirrhosis, fever, malaria, influenza, cancer, and menstrual disorders [84]. Our previous study provided a pioneering and important investigation of its potential anti-tumor effects. Specifically, the chemotherapeutic effects of an acetone extract of *B*. *scorzonerifolium* (BS-AE) demonstrated a distinct effect in terms of inhibiting the proliferation of A549 human lung cancer cells in vitro via the suppression of telomerase activity and the induction of apoptosis [85]. These highly promising results prompted us to further investigate the anticancer effects of *B*. *scorzonerifolium* derivatives, as well as to clarify their underlying mechanisms.

#### 5.2.2. Growth Arrest, Anti-Proliferation, and Apoptosis in Lung Cancer

**Figure 2:**
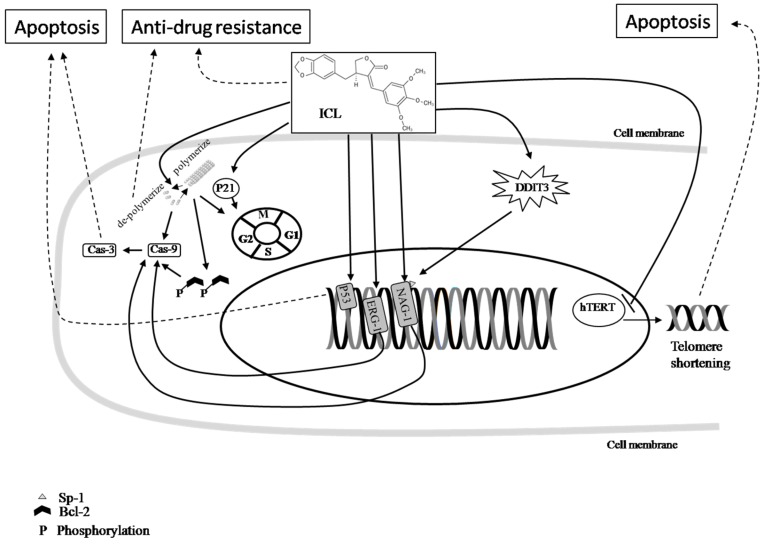
The cancer-fighting mechanisms of ICL. The effects of ICL in cancer therapy include impacts on cancer cell apoptosis, cell cycle arrest, telomerase inactivation, and anti-drug resistance. Symbol key: 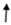 Pathway; 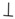 Inhibition; 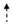 Final effect. Please see the content in Section 5.2.

In a subsequent study, we demonstrated the anti-cancer effects, both in vitro and in vivo, of one such compound, namely, isochaihulactone (ICL), which is isolated from the acetone extract of *B*. *scorzonerifolium*. Our results showed that ICL caused cell cycle arrest in the G2/M phase, the depolymerization of tubulin, and the activation of phospho-Bcl-2, caspase-3, and caspase-9 in a time- and concentration-dependent manner, as well as increasing p21 levels and the down regulation of the checkpoint proteins cyclin B1/cdc2 and cdc25, to induce apoptosis in A549 cells and suppress growth in A549 subcutaneous xenograft tumors in a dose-dependent manner without cytotoxic effects on normal lung fibroblast cells (Figure 2) [86].

#### 5.2.3. ICH induced Apoptosis through Nonsteroidal Anti-Inflammatory Drug-Activated Gene (*NAG-1*) Expression in Lung Cancer

To explore the mechanisms underlying ICH-induced growth arrest and apoptosis, we used oligodeoxynucleotide-based microarray screening to examine genetic changes before and after ICH treatment in a human lung carcinoma cell line, A549. Among various ICH-induced genes, we identified the early growth response gene-1 (EGR-1), a member of the zinc finger family of transcription factors that plays a significant role in cell growth and differentiation [87], and nonsteroidal anti-inflammatory drug-activated gene (*NAG-1*), which encodes a transforming growth factor-β like secreted protein and was initially characterized as a p53-regulated gene [88]. We found that ICH increased the mRNA and protein levels of EGR-1 and NAG-1, which were reduced by the mitogen-activated protein kinase 1/2 inhibitor. RNAi specific for NAG-1 inhibited ICH-induced apoptosis, and cells overexpressing NAG-1 had a greater apoptotic response to ICH through an Erk-dependent pathway involving the activation of EGR-1, suggesting that NAG-1 was a key molecule in the ICH induced apoptosis. This induction increased expression of NAG-1 that was p53-independent and Sp1-dependent, but phosphoinositide 3-kinase (PI3K) signaling was not involved. In vivo study supported that ICH increased the NAG-1 expression in an animal model [15].

#### 5.2.4. ICF induced Apoptosis in Prostate Cancer Cells by Activating NAG-1

By means of similar mechanisms, ICH has also been shown to cause cell cycle arrest at G2/M phase, induce growth inhibition, and commits the human LNCaP prostate cancer cells toward apoptosis by activating EGR-1 and NAG-1 through c-Jun N-terminal kinase (JNK)-dependent pathway instead of Erk way. Furthermore, ICH-induced cell death can be restored by siNAG-1 siRNA transfection. Our findings indicate that ICH is a potential antitumor compound for prostate cancer therapy [89].

#### 5.2.5. ICF Suppressed GBM Cells Growth by Increasing the Expressions of DNA Damage Inducible Transcript 3 (DDIT3) and NAG-1

In light of these promising effects of ICH against other types of cancer, our team has also recently begun to investigate the effects of ICH against GBM, with those investigations already yielding further promising results. Specifically, our data showed that ICH treatment disrupted the endoplasmic reticulum homeostasis in GBM cells by increasing DNA damage inducible transcript 3 (DDIT3) and NAG-1 expression. This led in turn to tumor cell cycle arrest at G2/M phase and apoptosis. The in vivo study showed that the ICH treatment caused a considerable reduction in tumor size, and the overexpression of DDIT3 and caspase-3 in the xenograft model [90].

#### 5.2.6. Anti-Chemoresistance

ICH in vitro is effective against the drug-resistant KB cell line (a subline of the ubiquitous Keratin forming tumor cell line HeLa), which overexpresses P-gp [91]. The ICH treatment in A549 cells and A549-T12 cells, which is a paclitaxel resistant/dependent A549 cell line with a α-tubulin mutation that may impair paclitaxel-driven tubulin polymerization [92], showed no significant difference, suggesting the role of ICH in anti-chemoresistance [91].

## 6. Conclusions

Although there are several attempts to improve the dismal prognosis of patients with cancer, including advanced surgical techniques and facilities, modified radiotherapy doses, schedules, and technique and the addition of new chemotherapy combinations, most have created little success. Only after a complete understanding of how molecular changes affect tumor growth rate and invasiveness, we may change to improve the dismal prognosis of patients with GBM.

Many herbs have been identified as possessing anti-cancer activity. Unlike chemotherapeutic regimes, these herbs, such as BP and ICH, cause a significant reduction of cancer size via a variety of molecules, such as Nurr77, DDIT3 and NAG-1 without harming healthy cells. Our pioneer works on highlight effects of these chemicals, including apoptosis, cell cycle arrest, penetration through the blood-brain barrier (BBB), Caspase activation and apoptosis, cell cycle arrest, anti-proliferation, senescence, telomerase inactivation, anti-chemoresistance, anti-invasion, anti-migration, and anti-dissemination against a number of cancers. Taken together, the results of these studies suggest that further investigations of BP and ICH as therapeutic compounds for treating various forms of cancer, including brain cancers, should be conducted.
